# Epileptic seizure focus detection from interictal electroencephalogram: a survey

**DOI:** 10.1007/s11571-022-09816-z

**Published:** 2022-05-18

**Authors:** Md. Rabiul Islam, Xuyang Zhao, Yao Miao, Hidenori Sugano, Toshihisa Tanaka

**Affiliations:** 1 Institute of Global Innovation Research, Tokyo University of Agriculture and Technology, Tokyo, Japan; 2 Department of Electrical and Electronic Engineering, Tokyo University of Agriculture and Technology, Tokyo, Japan; 3 Department of Neurosurgery, Epilepsy Center, Juntendo University, Tokyo, Japan; 4 Department of Electronic and Information Engineering, Tokyo University of Agriculture and Technology, Tokyo, Japan; 5 RIKEN Center for Brain Science, Saitama, Japan; 6 RIKEN Center for Advanced Intelligent Project, Tokyo, Japan; 7 Center for Precision Medicine, The University of Texas Health, San Antonio, USA

**Keywords:** Epilepsy, Interictal electroencephalogram (EEG), Seizure focus, Ripple and fast ripple, Phase amplitude coupling (PAC), High-frequency oscillation (HFOs), Interictal epileptiform discharges (IEDs), Neural network

## Abstract

Electroencephalogram (EEG) is one of most effective clinical diagnosis modalities for the localization of epileptic focus. Most current AI solutions use this modality to analyze the EEG signals in an automated manner to identify the epileptic seizure focus. To develop AI system for identifying the epileptic focus, there are many recently-published AI solutions based on biomarkers or statistic features that utilize interictal EEGs. In this review, we survey these solutions and find that they can be divided into three main categories: (i) those that use of biomarkers in EEG signals, including high-frequency oscillation, phase-amplitude coupling, and interictal epileptiform discharges, (ii) others that utilize feature-extraction methods, and (iii) solutions based upon neural networks (an end-to-end approach). We provide a detailed description of seizure focus with clinical diagnosis methods, a summary of the public datasets that seek to reduce the research gap in epilepsy, recent novel performance evaluation criteria used to evaluate the AI systems, and guidelines on when and how to use them. This review also suggests a number of future research challenges that must be overcome in order to design more efficient computer-aided solutions to epilepsy focus detection.

## Introduction

Epilepsy, one of the most common neurological disorders, can affect people of any age, race, or ethnic background. According to the latest study by the World Health Organization (WHO), approximately 65 million people worldwide are affected by epilepsy, and there are an estimated 2.4 million new cases each year (Giannakakis et al. 2014; Levesque et al. 2017; Stafstrom and Carmant 2015). Epilepsy, defined as repeated and unpredictable seizures, causes social impairment and a high risk of death (Fisher et al. 2014; Pati and Alexopoulos 2010). Childhood epilepsy also seriously impacts the development of the brain by reducing learning ability and mental growth. Epileptologists generally classify seizures as either focal or generalized based on abnormal brain activities (Ngugi et al. 2011; van Mierlo et al. 2014). To control epileptic seizures, epileptologists prescribe anti-epileptic drugs. When these medicines fail to control the seizures, surgical removal of the epileptic focus may be the patient’s best chance for seizure freedom.

According to the clinical guidelines related to epilepsy surgery, the epileptic seizure focus is the cortex area from which the seizures originate (Lüders et al. 2006). The identification and surgical removal of the focus must be resected (inactivated or completely disconnected) for complete seizure freedom. Standard diagnostic methods include inspection of seizure semiology, high-resolution magnetic resonance imaging (MRI), and EEG. The scalp EEG is a non-invasive method of recording electrical activity by placing electrodes on the scalp using the international standard 10–20 system (Paul 2018). It provides one of the promising ways to identify the epileptic seizure focus before surgical intervention. However, when epileptologists cannot determine an epileptic seizure focus using non-invasive methods, they indicate to use intracranial EEGs (iEEGs) with the implantation of intracranial electrodes during both interictal and ictal phases. Before the epileptic focus resection for complete abolition of seizures, the epilepsy surgeon should consider integrating the multi-channel intracranial electrodes to these “area of cortex” and recording iEEG signals until collecting enough data from habitual seizures to analyze. The epileptologists then need to inspect the multi-channel iEEG data to identify seizure onset zones (SOZs) from within the recorded cortex area. During these inspections, the epileptologists need to analyze and label all long-term multi-channel iEEG data, the manual detection of which is challenging and time-consuming. The success of the epileptic focus resection for seizure freedom depends on accurate detection of the seizure focus. A key to achieving good results with resection surgery is identifying and resecting the area that may cause seizures. Such areas are called epileptogenic zones (EGZs). Currently, there is no single, non-invasive test method that can identify those areas. Approximately 20–30 percent of patients suffer from recurrent seizures after surgery (Elsharkawy et al. 2011).

In the endeavor to design computer-aided diagnosis tools, both non-invasive and invasive iEEGs are promising procedures. Automatic detection of seizure focus is highly desired, as it would reduce the epileptologist’s workload and would, along with other tests, increase confidence in related medical decisions. Also, computer-aided and data-driven approaches may provide a way of revealing a mechanism of epileptogenesis.

For designing the computer-aided systems based on the different types of EEG modalities, Some recent studies used biomarkers, including high-frequency oscillations (HFOs)(Zijlmans et al. 2011; Jacobs et al. 2009; Urrestarazu et al. 2007), phase-amplitude coupling (PAC)(Guirgis et al. 2015; Motoi et al. 2018; Amiri et al. 2019), interictal epileptiform discharges (IEDs) (Staley and Dudek 2006; Elsharkawy et al. 2011) while others utilized feature-extraction methods (Sharma et al. 2015b; Akter et al. 2020a, 2019; Itakura and Tanaka 2017). In biomarker-related studies to identify epileptic seizure focus, the computer-aided solutions have combined the epilepsy biomarkers in EEG signals with advanced signal and machine-learning approaches. The epilepsy biomarkers in EEGs are essential for identifying the epileptic seizure focus within conventional clinical systems. However, from a machine learning perspective, the features extracted from EEG signals to characterize epileptic signals are also good candidates for identification of epileptic focus. To identify the epileptic focus from EEG signals, the recorded EEG signals are typically pre-processed using various signal processing methods, and features are extracted from the pre-process EEGs signals to represent in a compact form (Akter et al. 2020a, 2019; Hassan et al. 2019). The different studies introduced feature-extraction methods to design the computer-aided solutions for identifying epileptic focus (Sharma et al. 2015b; Akter et al. 2020a, 2019; Hassan et al. 2019; Itakura and Tanaka 2017). Based on the biomarker and feature-extraction related studies, there is a large diversity of research that have been explored in the last years to design AI-systems. Many new algorithms have been designed and explored in order to identify epileptic seizure focus.

However, there are few review papers that surveys works on automated machine learning-based focus detection methods. Recently, one review paper for focal and non-focal epilepsy localization (Hussein et al. 2018) has summarized the several studies with a variant of datasets to identify focal and non-focal channels. However, the main goal of most studies reported in their survey was to identify seizure patterns in continuous EEG signals instead of localizing focal and non-focal channels used in Bern Barcelona dataset (Andrzejak et al. 2001). For diverse research in the epilepsy, we would like to draw attention to the AI-solutions that different researchers have introduced for identifying the epileptic focus. In this paper, we survey the literature of the past decade to observe which new approaches have been investigated to design computer-aided automated systems and be the most efficient.

## Epileptic seizure focus

The epileptogenic zone (EGZ) is a conceptual definition of the area that causes seizures; resection of the EGZ halts them. When antiepileptic drugs fail to treat epilepsy, the patient is diagnosed with refractory epilepsy. One of the treatment approaches is removing the cortex’s possible area responsible for generating the abnormal neural activity resulting in epileptic seizure. In a study of surgical treatment of epilepsies, Lüders et al. (2006) categorized the EGZ as actual EGZ (commonly referred to SOZ) and potential EGZ. The study defined the SOZ as the cortex area from which actual clinical seizures are generated. The SOZ is most commonly localized by either scalp or intracranial EEG. In contrast, after resecting the SOZ, the seizure may continue from the other area of the cortex defined as the potential SOZ. This situation occurs when the surgery does not accomplish complete resection of the actual EGZ.

So far, the precise localization of the EGZ boundary cannot be characterized by any diagnostic methods. The most common methods to localize the SOZ are either scalp EEG or iEEG during both the interictal and ictal phases. Varied recent epilepsy research has suggested the use of ictal and interictal high-frequency activity ($\ge 80$ Hz) to localize the epileptogenic zone. In clinical practice, the SOZ is used to localize the boundary of the EGZ (Rosenow and Lüders 2001). In defining the possible boundary of the EGZ, the different cortical zones are measured using some standard diagnosis methods. The descriptions of these cortical zones and diagnostic tests are provided in Table 1. During the presurgical evaluation, epileptologists should consider these cortical zones when estimating the location and extent of EGZ (Talairach and Bancaud 1966; Lüders et al. 2006). To localize the possible EGZ, Talairach and Bancaud (1966) introduced three zones, namely a lesional, an irritative, and an epileptogenic zone. They reported that the SOZ was a reliable index of the location and extent of the possible EGZ and defined the SOZ as the epileptogenic zone.

**Table 1:** Descriptions of cortical zones and their clinical diagnosis tests. The diagnosis methods are: scalp electroencephalogram (EEG), interictal EEG (iEEG), magnetoencephalography (MEG), single photon emission computed tomography (SPECT), magnetic resonance imaging (MRI), and positron emission tomography (PET)

Cortical zones	Definition	Clinical diagnosis methods
Epileptogenic zone (EGZ)	Area of cortex responsible for generating epileptic seizures; resection yields seizure freedom	Scalp EEG and iEEG
Irritative zone	Area of cortex that generates interictal spikes	EEG and MEG
Seizure onset zone (SOZ)	Area of cortex from which clinical seizures originate	SPECT, scalp EEG, and iEEG
Epileptogenic lesion	Structural lesion that is related to the epilepsy	High-resolution MRI
Ictal symtomatogenic zone	Area of cortex that generates the seizure symptoms or signs	Ictal video recording
Functional deficit zone	Area of cortex that is not functioning normally in the interictal period	Neurological examination, Neuropsychological testing, Interictal PET and SPECT, Non-epileptiform EEG, and MEG

**Fig. 1:**
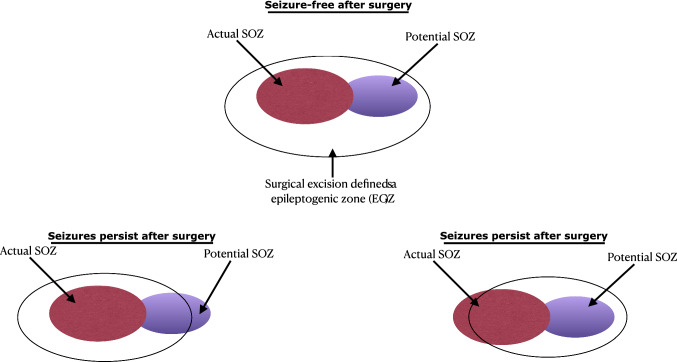
An example of SOZ and EGZ of a surgical resection with seizure-free or seizure-persistent surgical outcomes

Decades later, Lüders et al. (2006) enriched that concept by defining five cortical zones: the irritative zone (generates interictal spikes), seizure-onset zone (initiates clinical seizures), the symptomatogenic zone, the lesion, and the functional deficit zone. The detailed presurgical evaluation always requires defining these zones to understand the spatial relationship with the epileptogenic zone. Lüders et al. (2006) observed that, for some patients, complete resection of the SOZ does not lead to seizure freedom. After resecting the SOZ, the epileptic seizures were generated from the area in the brain closest to SOZ. Their study further dichotomized the SOZ into the actual and potential SOZ and suggested that the complete resection of both the actual and potential SOZ may be necessary for complete seizure cessation (see in Fig. 1). Therefore, epilepsy surgeons and epileptologists need to define the location and the extension of the EGZ by observing both SOZs before epileptic focus resection. Since the location and extension of the EGZ is a theoretical concept based on defining the actual and potential SOZs, clinical experts may only decide about the EGZ if the patient after surgery is seizure-free. Bancaud and Talairach (1992) also observed that, with patients with medically-intractable epilepsy, the SOZ is involved in a single region and interconnected with distinct regions.

Research is still ongoing regarding identification of the SOZ and the extent of brain area that should be considered for resection in order to achieve successful surgical treatment of epilepsy. In conventional clinical procedure for identification of the SOZ ( both actual and potential), the EEGs with multiple channels are recorded until enough data on habitual seizures has been collected. An epileptologist visually inspects the long-term multi-channel EEG signals to identify the electrodes with the possible SOZ that must be resected in order to achieve freedom from seizures. However, this process is challenging and time-consuming for epileptologists. Several studies have recently proposed computer-aided solutions based on either epilepsy biomarkers (HFOs, PAC, and IEDs) or the use of feature-extraction methods in EEGs. In the rest of this paper, we have reviewed the computer-aided designs (CAD) that might be utilized to identify the possible SOZ (Table 2).

**Table 2:** Summary of the datasets and goals of recent epilepsy research

Dataset name	No. of subjects	Electrode type	yEEG Type	Sampling frequency in Hz	Goal of the datasets
Bonn (Andrzejak et al. 2001)	5	Single-channel	Scalp EEG iEEG	173.61	Epileptic and non-epileptic patient detection
Flint Hill (Osorio et al. 2001)	10	Multi-channels	iEEG	240	Seizure detection
Freiburg (Winterhalder et al. 2003)	21	Multi-channels	iEEG	256	Seizure detection
n CHB-MIT$^{1}$ (Shoeb and Guttag 2010)	23	Multi-channels	Scalp EEG	256	Seizure detection
Epilepsiae (Ihle et al. 2012)	275	Multi-channels	Scalp EEG iEEG	250-2500	Seizure detection
TUSZ$^{2}$ (Obeid and Picone 2016)	315	Multi-channels	Scalp EEG	250	Seizure detection y
Bern-Barcelona (Andrzejak et al. 2012)	5	Binary-channels	iEEG	512	Epileptic focus detection

$^1$Children’s Hospital Boston-Massachusetts Institute of Technology

$^2$ TUH EEG Seizure Corpus

## Public datasets

Public epilepsy datasets can be roughly divided into two categories: scalp EEG and intracranial EEG. The first is used to detect abnormalities related to epileptic seizure and the second to determine epileptic focus location. What follow are some of the most commonly-used scalp EEG datasets.

- The Temple University Hospital (TUH) EEG Corpus (Harati et al. 2014), which is a large size and contains various sub-datasets, including abnormality detection, seizure detection, and artifact classification.
- The Children’s Hospital Boston (CHB-MIT) Scalp EEG Dataset (Goldberger et al. 2000) (Shoeb 2009), which is used for seizure detection.
- The University of Bonn EEG Dataset (Andrzejak et al. 2001) contains several different classes of data that are recorded from healthy volunteers and patients.
- Other common datasets include IEEG.org and the European Epilepsy Dataset.

**Fig. 2:**
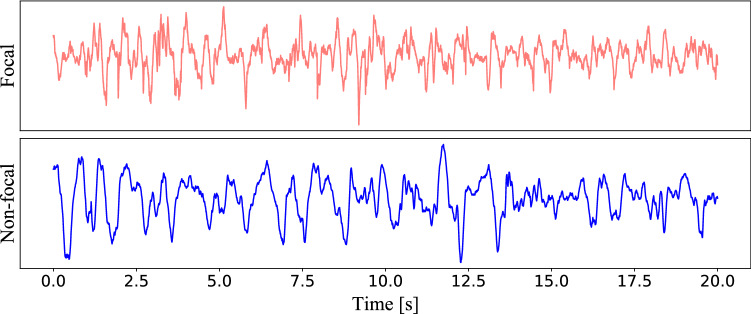
Samples of focal and non-focal iEEGs (Bern Barcelona Dataset)

Regarding the epileptic focus location problem, the most used public intracranial EEG dataset is the so-called Bern-Barcelona EEG dataset (Andrzejak et al. 2012). This dataset includes the iEEG signal recorded from five patients with long-standing pharmacoresistant temporal lobe epilepsy who are candidates for epilepsy surgery. iEEG signals are recorded by the AD-TECH device (Racine, WI, USA), which has a sampling rate of 512 Hz. The focal channel, which detected first ictal iEEG signal changes and judged throw visual by at least two clinical experts. The other channels are defined as non-focal channels. An example of focal and non-focal samples are shown in Fig. 2. The dataset includes 7500 samples of the focal and non-focal, respectively. Each sample is 20 seconds and is processed with the bandpass filter between 0.5 and 150 Hz by using fourth-order Butterworth filter.

This dataset has been extensively used as a benchmark in the literature; however, it also has the following drawbacks:

- It includes no information regarding locations of electrodes, which is essential for focal identification.
- Signals are provided as independent segments without patient labels.
- The highest frequency is limited to 150 Hz, even though recent neurological findings indicate that high frequency components (>100 Hz) are crucial to identify the epileptic focus.

## Bio-marker-based approach

### High frequency oscillation

**Fig. 3:**
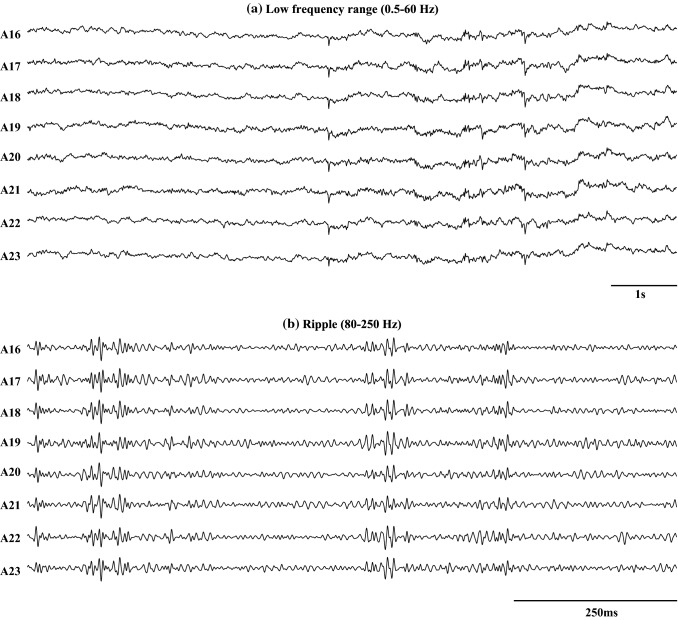
Example of filtered ECoG signal in the low frequency range of (0.5–60 Hz) and in the ripple band (80–250 Hz). A16–A23 are the channel names. **a** is the sub-figure of the filtered ECoG signal for 10s in a low frequency range of (0.5–60 Hz). **b** is the filtered ECoG signal for 1s in the ripple band (80–250 Hz)

High-frequency oscillations (HFOs >80 Hz) are short durational high gamma ripple and fast ripple activities in EEG signals, which are one of the biomarkers used to identify epileptic focus in conventional clinical settings (Zijlmans et al. 2011; Jacobs et al. 2009; Urrestarazu et al. 2007). In the human intracranial EEG signal, the frequency components of HFOs are greater than 80 Hz and can range up to 500 Hz. The sampling frequency needs to be at least 10 kHz to record HFOs in EEG signals. In general, the amplitude of HFOs varies between 10 and 1000 $\mu$V. An example of a filtered electrocorticography (ECoG) signal in the ripple band (80–250 Hz) and in a low frequency range (0.5–60 Hz) are shown in Fig. 3. To analyze the HFOs, extra care is necessary, as they might be corrupted by broad frequency components, such as spiking activity or other artifacts (Worrell et al. 2012). The HFOs are commonly categorized as ripples (100–250 Hz) or fast ripples (250–500 Hz) and are generally specific to the SOZ (Crépon et al. 2010). They are crucial for epilepsy surgery for removing entirely epileptic seizure focus required to produce seizure freedom (Lüders et al. 2006).

### Automated methods for HFOs detection

The methods of identification of HFOs are classified as manual, automatic, and a combination of the two approaches (Navarrete et al. 2016a). Manual detection, carried out by an expert or panel of experts, consists of visually monitoring a computer display. Experts must have a thorough understanding of electrophysiological recording and signal processing. Due to the current exponential growth in multi-channel measurement, manual inspection has become a difficult process, a situation that has undoubtedly increased the need for automatic detection. Therefore, to detect HFOs, several studies have proposed automated detectors over the past decade.The methodological advances for detecting HFOs in EEG signals occurred between 2002 and 2016 and Navarrete et al. (2016b) summarized in their study.

In the design of the HFO-based automated system, the main objective was to detect putative HFOs, including the pathological HFOs (pHFOs) and physiological-induced HFOs (nHFOs). The nHFOs, which were rejected based on manual observation by experts (Staba et al. 2002; Zelmann et al. 2012; Bénar et al. 2010; Liu et al. 2016) or implementation of complex methods based on machine-learning techniques (supervised or unsupervised) (Blanco et al. 2010; Matsumoto et al. 2013; Chaibi et al. 2014; Sciaraffa et al. 2020). There are many ways to extract putative HFOs from the desired frequency bands. The most common types of methods for automated detection are energy-thresholding-based methods (Staba et al. 2002; Zelmann et al. 2012; Bénar et al. 2010), including short-time energy (STE) (Staba et al. 2002), time line-length energy (Gardner et al. 2007), Hilbert transform envelope (HTE) (Crépon et al. 2010), the Montreal Neurological Institute (MNI) detector (Zelmann et al. 2012). Birot et al. (2013) combined three approaches, root mean square (RMS) amplitudes, line-length energy (LLE), and instantaneous frequency (IF), to identify HFOs. The above four methods for automated HFOs detection were also implemented in RIPPLELAB to assess performance (Navarrete et al. 2016a). Subsequently, other groups have proposed and tested algorithms, including Bumps modeling techniques (BMT) (Chaibi et al. 2013), estimation of power spectra by multitaper methods (Wang et al. 2014), RSM amplitudes in referential montage (Gliske et al. 2016), baseline detection by wavelet entropy (WE), and Hilbert envelope (Fedele et al. 2016), to monitor HFOs.

In order to deal with the classification problem of possible HFO events, the supervised and unsupervised classifiers such as clustering, Gaussian mixture model (GMM), support vector machines (SVM), random forest classifier (RF) are the useful method to differentiate the physiological and pathological HFOs (Blanco et al. 2010; Matsumoto et al. 2013; Chaibi et al. 2014; Sciaraffa et al. 2020; Varatharajah et al. 2018). However, classifiers’ unsupervised adaptation is much more difficult than supervised adaptation, as the class labels are unknown. The first examples of unsupervised approach (K-mediods clustering) were applied to detect HFO events proposed by Blanco et al. (2010). In modern clinical practice, the clinical translation of HFOs as a biomarker of EEGs has been largely restricted due to the difficulty in differentiating pathological HFOs from physiological HFOs (Jacobs et al. 2018; Fedele et al. 2019). Supervised automated detection techniques with SVM classifier were proposed by Matsumoto et al. (2013) for the differentiation between physiological and pathological HFOs. The line-length energy (LLE) method was used to a sliding 200-ms window with 50-ms overlap to detect the pathological HFOs (pHFOs) and physiological-induced HFOs (nHFOs) from background EEGs. A SVM classification was performed on the pHFO and nHFO using a rotating cross-validation approach by training the data for four patients and then classifying the remaining patient data. Chaibi et al. (2014) used six feature extraction methods to design an automated system. In the design of an automated system, a decision tree (DT) classifier has been trained with features of 200 events (100 events of background activity and 100 events of HFOs activity). In the testing phase, the 50-ms segments with with overlapping one sample were used to mark by 1 over time. They compared their system with five energy-based different approaches (STE (Staba et al. 2002), complex morlet wavelet (CMW) (Khalilov et al. 2005), Bumps modelling (BM) technique (Chaibi et al. 2013), and Hilbert (Zelmann et al. 2012)). Subsequently, different classification methods including linear discriminant analysis (LDA) (Jrad et al. 2015), SVM (Jrad et al. 2017), and radial basis function SVM (RBF-SVM) have been tested to detect HFOs in several studies (Sciaraffa et al. 2020). Some recent studies also try to adapt deep learning-based approaches for identifying the two kinds of HFOs in ripple and fast ripple iEEG (Medvedev et al. 2019; Zuo et al. 2019; Firpi et al. 2007). However, the above studies mainly focus on detecting HFOs in ripple and fast ripple iEEG data in a separate way rather than identifying the epileptic focus.

### Phase-amplitude coupling (PAC)

Many studies have recently reported on phase-amplitude coupling (PAC) analysis of epileptic EEG. Nariai et al. (2011) have observed that the interictal HFOs at 80–200 Hz within the seizure onset zone (SOZ) are tightly locked to the phase more than 3 Hz of slow-wave. On the contrary, ictal HFOs are tightly locked to the phase less than 1 Hz of slow-wave. Guirgis et al. (2015) investigated that low-frequency oscillation (LFO)-modulated HFOs might be employed to identify regions of interest (ROI) in extratemporal lobe patients. Moreover, Amiri et al. (2016) found that PAC was more substantial in the SOZ than outside of it, and PAC was higher in spiking channels outside the SOZ than in normal brain areas. Another study found that the HFOs coupled to phases of theta band can be used to identify SOZ (Amiri et al. 2019). Weiss et al. (2016) examined the ripple amplitude, which was significantly modulated by a phase of epileptic EEG spike with the SOZ. Last, Motoi et al. (2018) reported in their study that PAC strength is different between the ripple and a phase of 3–4 Hz.

PAC can be simply explained as the coupling on high-frequency amplitude modulated by low-frequency phase, which reveals the coupling phenomenon between brain rhythms, especially between slow-waves and fast-waves, like that seen in high-frequency oscillation (HFO). In many studies, the PAC strength is quantified using the mean vector length modulation index (MVL-MI) (Canolty et al. 2006). Specifically, there are three steps to calculate the PAC strength through the MVL-MI approach. Firstly, defining a complex component as

1 $$\begin{aligned} z(t)=A_{amp}(t)e^{i\phi _{pha}(t)}, \end{aligned}$$

where the high-frequency amplitude and low-frequency phase are denoted as $A_{amp}(t)$ and $\phi _{pha}(t)$, respectively. Secondly, defining the raw modulation index as

2 $$\begin{aligned} MI_{raw}=\left| \overline{z(t)}\right| . \end{aligned}$$

Thirdly, a surrogate data approach is introduced to z-scored the $MI_{raw}$ by using the mean (denoted as $\mu$) and standard deviation (denoted as $\sigma$) of 100 surrogate data. The final modulation index can then be defined as

3 $$\begin{aligned} MI=\frac{MI_{raw}-\mu }{\sigma }, \end{aligned}$$

where the *MI* is used to evaluate PAC strength.

**Fig. 4:**
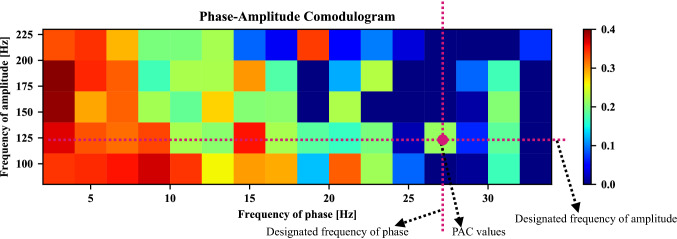
Example of phase-amplitude comodulogram

Hereafter, in order to graphically display a series of PAC values of different frequencies, the phase-amplitude comodulogram approach is introduced. A simple example is shown in Fig. 4, the frequency of phase is represented in the horizontal axis, while the frequency of amplitude is displayed in the vertical axis. The PAC value between the designated frequency of phase and the amplitude’s designated frequency is exhibited using a pseudocolor plot in which a hot color denotes high coupling strength.

In more recent studies, some machine-learning methods have been applied to identification of the SOZ based on PAC features. Varatharajah et al. (2018) proposed the support vector machine (SVM) approach with PAC features to detect SOZ. Elahian et al. (2017) employed logistic regression technology based on the PAC values of ictal ECoG recordings to identify SOZ electrodes. In the last decade, it has become popular to combine promising features and AI-based models for SOZ detection. Table 3 summarizes the literature about PAC analysis for SOZ localization that was published between 2015 and 2019. ETLE, MTLE, and mTLE represent extratemporal lobe epilepsy, mesial temporal lobe epilepsy, and mesiotemporal lobe epilepsy, respectively. $MI_{30-450 Hz \& 0.5-4 Hz}$ denotes PAC strength between the amplitude of 30–450 Hz and the phase of 0.5–4 Hz.

**Table 3:** Summary of PAC analysis for SOZ localization since 2015 to 2019

Authors	Dataset	EEG type	Significant PAC range
Guirgis et al. (2015)	7 patients with ETLE	iEEG	$MI_{30-450 Hz \& 0.5-4 Hz}$
Amiri et al. (2016)	25 consecutive epileptic patients (Montreal Neurological Institute and Hospital)	Scalp EEG	$MI_{30-260 Hz \& 0.3-13 Hz}$
Weiss et al. (2016)	12 patients with MTLE (UCLA Seizure Disorder Center)	iEEG	PAC between ripple amplitude and epileptiform spike phase
Elahian et al. (2017)	10 patients with epilepsy (Le Bonheur Children’s Hospital)	ECoG	$MI_{80-150 Hz \& 4-30 Hz}$
Motoi et al. (2018)	123 patients with drug-resistant focal epilepsy (Children’s Hospital of Michigan and Harper University Hospital in Detroit)	ECoG	$MI_{150-300 Hz \& 3-4 Hz}$
Varatharajah et al. (2018)	82 patients with focal epilepsy (Mayo Clinic, Rochester, MN)	iEEG	$MI_{65-115 Hz \& 0.1-30 Hz}$
Amiri et al. (2019)	18 patients with mTLE (Montreal Neurological Institute and Hospital)	iEEG	$MI_{HFOs \& 4-8 Hz}$

### Interictal epileptiform discharges (IEDs)

**Fig. 5:**
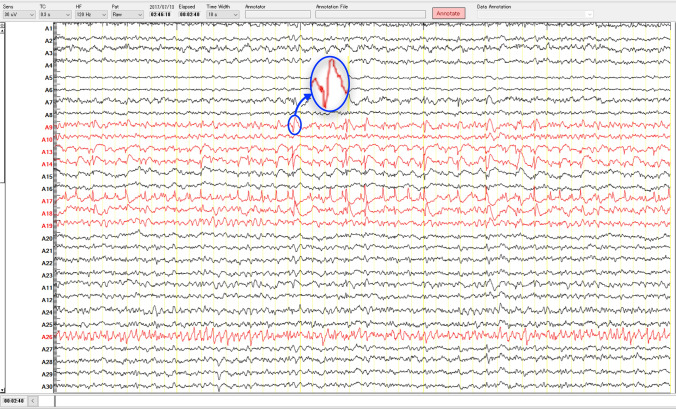
An example of IEDs (blue circle) in the SOZ channels (red color) as labeled by clinical experts. The iEEG data with a sampling rate of 2 kHz was measured in Juntendo University Hospital, Tokyo, Japan. (Color figure online)

Interictal epileptiform discharges (IEDs), which are generated from the irritative zone, are another important biomarker of epilepsy surgery that is used to localize or extend epileptic focus. The IEDs are morphologically classified as spikes, sharp waves, poly-spike complexes, or as a multiple-spike complex (Noachtar et al. 1999; Staley and Dudek 2006; De Curtis and Avanzini 2001). The IEDs are clearly distinguished from background activity through visual inspection of long-term multichannel EEGs by EEG experts. Figure 5, which was collected from the Juntendo Hospital, shows an example of IEDs (blue circle) in the SOZ channels. According to the definition set by the International Federation of Societies for Electroencephalography and Clinical Neurophysiology (IFSECN) (Deuschl 1999; De Moraes and Callegari 2014), the spikes have a duration between 20 and 70 ms, while sharp waves last between 70 and 200 ms. The spike-and-wave complexes are defined as spikes followed by slow waves that have a duration from 150 to 350 ms. However, standard modalities to observe the IEDs from background activity are either EEG or MEG recorded from epilepsy patients (Lüders et al. 2006). Since our review study is primarily concentrated on detecting epileptic focus using EEG signals, most of the algorithms that have been introduced in this section based on EEG spike detection methods. It is important to note that detection of IEDs in the EEG signals is one of the most difficult tasks for epileptologists due to the variation of spikes morphology and their similarities to artifacts (Wilson and Emerson 2002; Oikonomou et al. 2007) such as muscle activity and eye blinks.

In automated IED-based detection studies, many detection algorithms have been proposed based on single and multichannel EEG approaches (Oikonomou et al. 2007). The reviews that study automatic detection of IED spikes have been summarized in several review papers over the last four decades. (Pardey et al. 1996; Wilson and Emerson 2002; Abd El-Samie et al. 2018; Halford 2009; De Moraes and Callegari 2014). Several studies have categorised the existing detection methods into mimetic analysis (Gotman and Gloor 1976; Exarchos et al. 2006; Boos et al. 2011; Ji et al. 2011b; Halford et al. 2013), template matching (Stevens et al. 1972; Fischer et al. 1980; Kalayci and Ozdamar 1995; Ji et al. 2011a; Nonclercq et al. 2012), parametric approaches (Oikonomou et al. 2007; Durka 2004; Acır and Güzeliş 2004; Radmehr and Anisheh 2013),and power spectral analysis (Adjouadi et al. 2004a; Barlow 1980). The mimetic approaches to detection of IEDs from background activity split the long-term iEEG signals into half-waves based on amplitude extrema; two half-waves create the triangular shape of an epileptic transients with opposite directions (Halford 2009). In identifying the waves related to IEDs, each wave is examined against the set of predetermined criteria provided by EEG experts. These criteria were created based upon the distinctive attributes of IEDs, namely area, slope, amplitude, duration, sharpness, and dominant frequency. Most of the detection methods are based on thresholding (Garg and Kohli 2013; Juozapavicius et al. 2011; Xu et al. 2007, 2006) or on complex algorithms with a classifier (Exarchos et al. 2006; Saastamoinen et al. 1998; Liu et al. 2013). Nevertheless, as many studies have noted, the traditional morphological definition can be changed, particularly during long-term iEEG recording due to eye blinks, vertex waves, electrode artifacts, and movement artifacts (Adjouadi et al. 2004a; Nakamura et al. 2001). To improve the performance of the mimetic approach, some studies have utilized pre-processing via the the Walsh operator, Fourier transform (FT), or incorporation of additional channels, such as electrocardiogram (ECG), electrooculogram (EOG), or electromyogram (EMG) signals (Glover et al. 1989; Black et al. 2000; Nakamura et al. 2001).

Biomedical signals like EEGs are mostly non-stationary, which implies a time-varying frequency spectrum. For such signals, either an adaptive or a non-adaptive model can be used to identify epileptiform events (Isaksson et al. 1981; Pardey et al. 1996; Oikonomou et al. 2007). Some attempts have been made to describe EEG signals through use of a mathematical model characterized by a set of parameters. Methods, such as autoregressive (AR), moving average (MA), and autoregresssive moving average (ARMA), can be utilized to create time-invariant models for EEG signals. Under the assumption of non-stationarity epileptiform events, Kalman’s filtering approaches have also been adapted to enhance EEG signals before spike detection (Oikonomou et al. 2007). However, these methods are prone to false detections due to contamination or corruption by a set of artifacts (Kalayci and Ozdamar 1995; Azami and Sanei 2014; Oikonomou et al. 2007). For recent advances in AI-based methodologies, some authors have combined advanced signal processing approaches and classification with the above techniques to isolate IED spikes from background signals. The wavelet transform is a powerful tool in signal processing that has led to numerous biomedical applications, and several studies have focused on its use in neural networks to detect EEG spikes (Halford et al. 2013; Song and Zhang 2013; Kalayci and Ozdamar 1995; Gopan et al. 2013; Chavakula et al. 2013). Azami and Sanei (2014) used smoothing techniques with noisy scenarios, namely, discrete wavelet transform (DWT), Kalman filter (KF), singular spectrum analysis (SSA), Savitzgy-Golay (SG) filter, and empirical mode decomposition (EMD) for improving performance of AI-solution.

Many studies have focused on developing template-matching approaches (Lodder et al. 2013; Ji et al. 2011b, a; El-Gohary et al. 2008; Hese et al. 2008) in which the EEG experts visually select a few spikes from a set of EEG signals. These spikes were averaged to generate a template, and time-shifted correlation approaches were applied to determine the similarity between each segment and the template. Automated techniques to identify the spikes include simply checking the threshold or the use of algorithms, such as clustering, SVM, or more complex classifiers (Lodder et al. 2013; Ji et al. 2011b, a; El-Gohary et al. 2008; Hese et al. 2008). A frequency-domain analysis can be an important tool in the analysis of epileptic spikes. The most common power spectral analyses are used in epileptic spike detection, including Fourier transform (FT) (Logesparan and Rodriguez-Villegas 2011), Hilbert transform (HF) (Witte et al. 1991; Feucht et al. 1997), Walsh transform (WT) (Adjouadi et al. 2004b), and more advanced methods, such as wavelet analysis (Özdamar and Kalayci 1998; Goelz et al. 2000). In order to develop a more accurate detection system, some studies have proposed decomposition methods to divide the EEG signals into narrow subbands (Bourien et al. 2004; Pietilä et al. 1994; Witte et al. 1991), such as delta, theta, alpha, beta, and gamma, which allows for accurate estimation of the distinctive attributes of interictal spikes. Periodicity-based methods may be performed because EEG signals may exhibit some periodicity in the presence of IED spikes (Fürbass et al. 2015; Herta et al. 2015; Koren et al. 2015). In recent years, deep learning techniques like convolutional neural networks (CNN) have exhibited superior performance regarding detection of IEDs in EEG signals, especially when trained on large databases (Fukumori et al. 2019). However, because the detection of IEDs is critical to extending the boundary of SOZ, IED-related research has mainly focused on identifying IEDs rather than on localization of epileptic focus detection (Table 4).

**Table 4:** Different types of feature-extraction methods used to detect epileptic focus

Information theoretic methods	Statistical methods
Sample entropy (SE), permutation entropy (PE), delay permutation entropy (DPE), approximate entropy (APE), fuzzy entropy (FzE),y Reny’s entropy (REN), Shannon entropy (SE), Tsallis entropy (Ts), phase entropy (S1 and S2), wavelet entropy (WE), k-nearest neighbors- entropy (kNNE), centered correentropy (CCE), Stein’s unbiased risk- estimate entropy (SUREE), log-energy entropy (LEE), multi-variate entropy (MVE)	Mean, variance (Var), standard deviation (SD), coefficient of variation, mean absolute value,modified mean absolute value (MMAV), MMAV2, fluctuation index, log detector median frequency (MDF), mean frequency (MNF), katz fractal dimension (KFD), fractal dimension (FD), skewness, kurtosis, different types of quartile: (Q1, Q3, interquartile range), largest lyapunov exponent (LLE), root mean square (RMS), band power (BP), zero crossing (ZC), Hjorth parameter: (activity, mobility, and complexity), teager energy, 1st and 2nd derivative: (mean, SD, var), recurrence qualitative analysis (RQA): mean diagonal line length (MDLL), laminarity (LAM), trapping time (TT), longest vertical line (LVL), longest diagonal line (LDL), recurrence times (RT), Kolmogorov Complexity (KC), Lempel-Ziv complexity (LC)

## Statistical feature extraction

Apart from the above biomarker-related, computer-aided design (HFOs, PAC, and IEDs), the statistical features have, over the last couple of years, also been used as an engineering markers for the identification of possible SOZ. It is important to note that the iEEG is the biosignals, and such biosignals contain useful information of diseases about biological systems. However, it is not easy to identify that information solely through visual observation of the raw signals. EEG signals have a significantly low signal-to-noise ratio (SNR) and are frequently corrupted during recording by external- and internal-source artifacts (Islam 2015; Islam et al. 2018). The presence of artifacts and noise poses a significant challenge to analysis of recorded signals, and thus, to useful information extraction or classification. The direct use of machine-learning approaches to biosignals without pre-processing or feature extraction may lead to a decrease in sensible decisions by the automated system. Pre-processing, signal processing, and machine-learning approaches were combined with a standard computer-aided design to deal with EEG artifacts and to extract and decode the relevant features of EEG signals. In an epileptic focus detection system, the most commonly used pre-processing step was division of the long-term EEG data into a set of segments or frames. The segmentation of that long-term EEG data was dependent upon data size, system performance, and the direction of clinical experts (Akter et al. 2020a, 2019; Hassan et al. 2019).

Feature-extraction methods can be applied to the segments to extract the practical features. To implement epileptic focus detection, several studies since 2020 have suggested utilizing information-theoretic and statistical approaches. The statistical features have been shown to be particularly useful at detecting the epileptic focus in different studies. After extracting features from iEEG signals, the next step is to classify the normal and epileptic events using a classifier, such as k-nearest neighbor (kNN), linear discrimination analysis (LDA), support vector machine (SVM), or an implementation of a complex algorithms (i.e., neural networks (NN)).

For the design of computer-aided solutions to the problem of identifying epileptic seizure focus, several epilepsy research exploited Bern-Barcelona iEEG dataset, which was considered benchmark (Table 5). The Bern-Barcelona iEEG dataset consists of a pair of focal and non-focal signals recorded in the epileptogenic and non-epileptogenic zones of the brain. In order to observe the focal and non-focal channels in iEEG, Zhu et al. (2013) first introduced the delay permutation entropy (DPE) method with an SVM classifier. Experimental results show that the DPE index of focal channels was significantly lower than that of the non-epileptogenic hemisphere. Sharma et al. (2014) proposed sample entropy as a feature and the least square SVM (LS-SVM) classifier was used to discriminate focal and non-focal features. The mean and standard deviation (SD) were also used to differentiate between the two channels (Deivasigamani et al. 2016). Three machine-learning approaches, including kNN, fuzzy Sugeno classifier (FSC), and LS-SVM, have been trained using the wavelet entropy features (Sharma et al. 2015b). The features were Shannon, Renyi’s, Tsallis, fuzzy, permutation, and phase entropies. However, the nature of EEG and ECoG signals is very complex, non-stationary, and time-dependent. Due to the non-stationary properties of EEG data, the linear feature-extraction methods do not adapt perfectly to the EEG signals. Therefore, several studies proposed decomposition methods to improve the performance of the system, including wavelet transformation (WT) (Gupta and Pachori 2020), wavelet filter-bank (WFB) (Sharma et al. 2017), tunable-Q wavelet transformation (TQWT) (Sharma et al. 2017), flexible analytic wavelet transform (FAWT) (Gupta et al. 2017), and Empirical WT (EWT) (Bhattacharyya et al. 2018). A comparison study with Bern-Barcelona dataset using time-domain multiband analysis, including EMD and bivariate EMD (BEMD) (Rilling et al. 2007; Subasi et al. 2019), was reported by Itakura and Tanaka (2017) and (Bhattacharyya et al. 2018). Recently, new developments in computer-aided systems have suggested 23 feature-extraction methods that might be used to extract features from EEG signals (Acharya et al. 2019). The feature-extraction methods used to design those systems were different types of entropies, fractal dimension (FD), RMS, Hjorth parameters, Hurst exponent, KLC, LZC, and QRA. Detailed descriptions of the feature-extraction methods are provided in Table 4. To correctly detect focal and non-focal EEG signals, LS-SVM was adapted with feature ranking based on student t-test. To demonstrate the efficacy of features, some authors have investigated the several kinds of classifiers, including adaptive neuro fuzzy inference system (ANFIS), probabilistic neural network (PNN), naïve bayes (NB), non-nested generalized exemplars classifier (NNge), best first decision tree (BFDT), and radial basis function kernel-SVM (RBF-SVM). Though most of the research performed since has used the Bern-Barcelona dataset to design computer-aided systems capable of detecting epileptic seizure focus, some have explored different feature-extractions and machine-learning methods. Algorithmic progress has been limited due to the lack of a standard multi-channel benchmark iEEG datasets. Nevertheless, how the implementation of any of these methods will influence epileptic focus detection for real clinical applications is not yet clear.

**Table 5:** Summary of epileptic seizure focus detection based on statistical feature extraction from iEEG signals. Each of these methods was published 2013–2020. All methods were tested with the Bern-Barcelona dataset (iEEG, 0.5–150 Hz)

Authors	Features	Classifier	Evaluation
Zhu et al. (2013)	Information theoretic features	SVM	ACC: 84%
Sharma et al. (2014)	EMD+Information theoretic features	LS-SVM	ACC: 85%
Sharma et al. (2015a)	EMD+Information theoretic features	LS-SVM	ACC: 87%; Sen: 90%; Spe: 84%
Sharma et al. (2015b)	DWT+Information theoretic features	PNN, kNN, FSC, LS-SVM	yACC: 84%; Sen: 84%; Spe: 84%
Deivasigamani et al. (2016)	DTCWT+ Statistical methods	ANFIS	ACC: 99%; Sen: 98%; Spe: 100%
Das and Bhuiyan (2016)	EMD-DWT+ Information theoretic features	kNN	Acc: 89.40%; Sen: 90.70%; Spe: 88.10%
Sharma et al. (2017)	Wavelet FB+Information theoretic features	SVM	ACC: 94.25%; Sen: 91.95%; Spe: 96.56%
Sharma et al. (2017)	TQWT+Information theoretic, statistical features	SVM	ACC: 95%;
Gupta et al. (2017)	FAWT+Information theoretic features	kNN, LS-SVM	ACC: 94.41%; Sen: 93.25%; Spe: 95.57%
Bhattacharyya et al. (2017)	TQWT+ Information theoretic features	LS-SVM	Acc=84.67%; Sen=83.86%; Spe=85.46%
Sriraam and Raghu (2017)	Information theoretic, statistical features	SVM	ACC: 92.15%; Sen: 94.56%; Spe: 89.74%
Arunkumar et al. (2017)	Information theoretic features	NB, SVM, kNN, NNge, BFDT	ACC: 98%; Sen: 100%; Spe: 96%
Itakura and Tanaka (2017)	BEMD+Information theoretic features	RBF SVM	ACC: 86.89%
Chen et al. (2017)	DWT+ Statistical features	RBF SVM	ACC: 88%
Bhattacharyya et al. (2018)	EWT+ Statistical features	LS-SVM	yACC: 90%; Sen: 98%; Spe: 92%
Acharya et al. (2019)	Statistical features	LS-SVM	yACC: 87.93%; Sen: 89.97%; Spe: 85.89%
Dalal et al. (2019)	FA-WT+Statistical features	RELS-TSVM	ACC: 90.2%
Subasi et al. (2019)	EMD+DWT+WPD features	RF	ACC: 99.92%
Gupta and Pachori (2020)	WT+Information theoretic features	LS-SVM	ACC: 95.85%; Sen: 95.47%; Spe: 96.24%
Sharma et al. (2020)	Statistical features	SVM	ACC: 99%

Recently neurology hospitals measure ECoG with a high sampling frequency (20 kHz) and high-density electrode grids (multi-channels) in order to localize the epileptic seizure focus. Most recently, the connectivity and synchronization processes of the epileptic brain have been analyzed in different studies (Klimes et al. 2015; Warren et al. 2010; Antony et al. 2013). In general, the epileptic brain is distinguished by increased neuronal synchrony, and the epileptic focus is often functionally disconnected from the surrounding areas (Warren et al. 2010; Antony et al. 2013). The energy variation of the SOZ is significantly higher than that of the non-epileptic zone of the ripple (80–250 Hz), and fast ripple (250–600 Hz) bands (Klimes et al. 2015; Warren et al. 2010; Antony et al. 2013). In particular, the repetitive waveform pattern called the high-frequency oscillations (HFOs) consists of ripple, and fast ripple are the valid frequency bands to localize the epileptic seizure focus (Jacobs et al. 2009; Zijlmans et al. 2011; Urrestarazu et al. 2007). Based on these recent findings related to epileptic focus and clinical application, the computer-aided solutions that utilize feature-extraction methods encompass several open research questions that deserve to be addressed. The Bern-Barcelona dataset does not support the neurological and clinical biomarker evidence that is collected through use of ripple (80–250 Hz) and fast ripple (250–600 Hz) bands.

In a clinical situation, the number of SOZ and non-SOZ channels in the cortex area depends on the patient, which creates an imbalanced problem. In our survey, only two recent studies related to the design of a computer-aided solution, those proposed by Akter et al. (Akter et al. 2020a, b) addressed these unresolved questions. In their first study, the information-theoretic entropy features were investigated in order to design a computer-aided solution for identification of the SOZ channels of patients with drug-resistant epilepsy. The same research team recently observed the statistical features of high-frequency bands of interictal iEEG that efficiently identify the epileptic focus channels. To assess the possibility of building a patient-independent design (PID), they compared their proposed patient-dependent system with a patient-independent system for localizing SOZ channels through use of the optimal classifier LGBM. Ultimately, their system to extract features from high-frequency iEEG signals and thereby detect epileptic focus completely satisfies neurological hospital measurement standards and complements recent research findings related to epileptic seizure focus. However, many investigations are still required to improve the system’s performance and usability (Akter et al. 2020b).

## Neural networks: end-to-end approach

With the rapid growth of neural-network-based methodologies in recent decades, the computer-aided designs that use these methods have been introduced to assist clinical experts in the diagnosis and treatment of epilepsy.

### Structure of neural network

The most important aspects of a neural network in this context are the convolutional layer, recurrent layer, fully connected layer, pooling layer, activation function, and batch normalization, among others.

- The convolutional layer consists of a set of learnable filters (or kernels), each of which has a small receptive field. Dot product (inner product) is performed between the filter weights and region in the input data. The output of the convolutional layer is called the feature map; its depth can be controlled by the number of filters. The stride is set to control how much the filter convolves across the input data.
- The recurrent layer operates within the cyclical nature of data input and output; each output builds upon the one before it. The RNN with a tanh activation function can be defined as:
  4 $$\begin{aligned} h_{t} = \tanh (W_{x}x_{t} + W_{h}h_{t-1} + b), \end{aligned}$$
  where $x_{t}$ is the input data of the time *t*, and $h_{t}$ and $h_{t-1}$ are the hidden states of the time *t* and $t-1$, respectively. $W_{x}$ and $W_{h}$ are the learnable parameter matrices used for learning input data and hidden state, respectively. *b* is the parameter vector of the bias.
- The pooling layer performs the downsampling operation for the input data, which can lower the calculation complexity and prevent overfitting. Some commonly-used pooling operations are max pooling and average pooling, both of which partition the input data into a subregion set. For each subregion, the output is either the maximum or the average value.
- The fully connected layer is used to compute the class scores in the last layer. A one-dimensional feature vector immediately precedes this layer and functions as its input. In a fully-connected layer, each neuron is connected to all the numbers in the previous volume, which is identical to the traditional multi-layer perceptron neural network.
- The activation function is a non-linear mathematical operation between the current neuron and its output to the next layer. The definitions of some of the commonly-used activation functions are as follows: $\text{ Sigmoid } (x) = \frac{1}{1 + e^{-x}}$, $\tanh (x) = \frac{e^{x} - e^{-x}}{e^{x} + e^{-x}}$, and $\text{ ReLU }(x) = \max (0, x)$, in each of these, x is the input variable.
- The batch normalization layer is used for re-centering and re-scaling the input data, which stabilizes the neural networks by allowing for faster convergence. The calculation includes two steps. The first calculates the mean $E(\cdot )$ and variance $\text{ Var }(\cdot )$ of a batch data. In the second step, each sample is centered by subtracting the mean and dividing it by the standard deviation: $y = \frac{x - E(x)}{\sqrt{\text{ Var }(x)}}$, where *x* is the input variable and *y* is the normalized result.

Two typical neural network models (CNN and RNN models) are often used to process epilepsy brain signals. The CNN model can capture the waveform features of the EEG signals through the convolution operation. As one of the most widely-used convolutional neural network is VGG model (Simonyan and Zisserman 2015), the CNN achieved first and second places in the localization and classification tracks, respectively, in the Large Scale Visual Recognition Challenge (ILSVRC) of 2014. The model structure is shown in Table 6. Due to the effectiveness of this model in the field of machine-learning approaches, several studies have recently proposed a variant of one-dimensional convolution models for epilepsy signal processing.

**Fig. 6:**
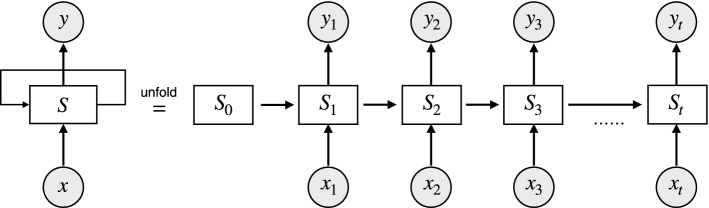
An unrolled recurrent network. $x_{t}$ and $y_{t}$ are the input and output at time *t* for each neuron. The input of each neuron contains not only the current input but also the output of the previous neuron

Unlike the other neural networks, the RNN uses an internal memory unit to process the result of the previous neural unit. However, the brain signals are time sequence data that depend upon causal relationships with the data before and after. Therefore, the RNN model is suitable for processing brain signals. A basic unit of the RNN model is shown in Fig 6.

**Table 6:** VGG Model Structure (The depth of the model increases from the left (A) to the right (E), the ReLU activation function is not shown for brevity.)

VGG Model Structure					
A	A-LRN	B	C	D	E
Input: 224*224 RGB Image					
Conv3-64	Conv3-64	Conv3-64	Conv3-64	Conv3-64	Conv3-64
	LRN	Conv3-64	Conv3-64	Conv3-64	Conv3-64
*Maxpool*					
Conv3-128	Conv3-128	Conv3-128	Conv3-128	Conv3-128	Conv3-128
		Conv3-128	Conv3-128	Conv3-128	Conv3-128
*Maxpool*					
Conv3-256	Conv3-256	Conv3-256	Conv3-256	Conv3-256	Conv3-256
Conv3-256	Conv3-256	Conv3-256	Conv3-256	Conv3-256	Conv3-256
			Conv3-256	Conv3-256	Conv3-256
					Conv3-256
*Maxpool*					
Conv3-512	Conv3-512	Conv3-512	Conv3-512	Conv3-512	Conv3-512
Conv3-512	Conv3-512	Conv3-512	Conv3-512	Conv3-512	Conv3-512
			Conv3-512	Conv3-512	Conv3-512
					Conv3-512
*Maxpool*					
Conv3-512	Conv3-512	Conv3-512	Conv3-512	Conv3-512	Conv3-512
Conv3-512	Conv3-512	Conv3-512	Conv3-512	Conv3-512	Conv3-512
			Conv3-512	Conv3-512	Conv3-512
					Conv3-512
*Maxpool*					
FC-4096					
FC-4096					
FC-1000					
Softmax					

### Methods based on neural networks

**Fig. 7:**
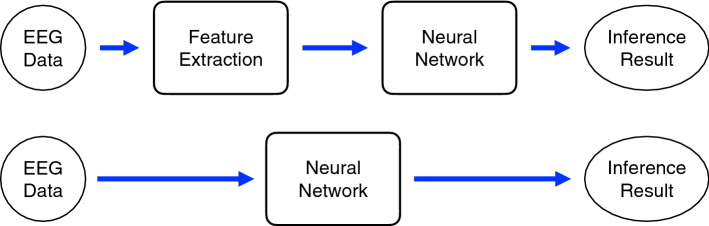
A common neural network models. The top panel shows a traditional machine-learning model, which includes feature extraction and classification. The bottom panel illustrates the end-to-end machine-learning model that classifies directly from the input data

In recent years, neural network methods have been applied to assist with diagnosis and treatment of epilepsy. The use of neural networks is mainly divided into two types. In the first type, feature extraction is performed through an algorithm, and then the neural network is used as a classifier. In the second type, feature extraction and classifier are all done by the neural network in an end-to-end model. Two commonly-used neural network models are show in Fig. 7.

Sui et al. (2019) used short-time Fourier transform (STFT) method to extract time-frequency features of the EEG signals. The obtained features were used as an input to the CNN model. Subathra et al. (2020) used the fast Walsh-Hadamard transform (FWHT) method to analyze the EEG signals in the frequency domain and decomposed them into the Hadamard coefficients. Five different nonlinear features were extracted from the decomposed Hadamard coefficients: approximate entropy (ApEn), log-energy entropy (LogEn), fuzzy entropy (FuzzyEn), sample entropy (SampEn), and permutation entropy (PermEn).To discriminate focal and non-focal channels, the artificial neural network (ANN) was used as a classifier. Siddharth et al. (2019) decomposed the EEG signals by using sliding mode-singular spectrum analysis (SM-SSA). Next, the classifier was designed by combining the sparse-autoencoder (SAE) hidden layer and the radial basis function neural network (RBFN). By using multiple filters and entropy (Zhao et al. 2018), features matrices were extracted from EEG signals, then focal and non-focal features were classified based on CNN method. San-Segundo et al. (2019)proposed a convolutional and fully-connected layers as classifier using a variant of multi-band decomposition analysis such as FT, WT, and EMD. Gagliano et al. (2019) introduced bispectrum analysis as the signal features; it resulted in a two-dimensional mapping of nonlinear interactions between the various frequency components of a time series. Last, the classification was implemented by using long-short-term memory (LSTM). Recently, by taking into account the feature interaction information, Zhao et al. (2021) integrated the tensor fusion strategy into deep CNN method for identifying epileptic focus.

The end-to-end method refers to training a possibly complex learning system represented by a model and avoiding the traditional feature process. Daoud and Bayoumi (2019) automated and integrated extracted features and the classification processes by using both a deep convolutional auto-encoder and multi-layer perceptron. Due to the EEG signal being one-dimensional data, Li et al. (2019) used a one-dimensional convolutional neural network model to perform the feature extraction and classification functions at the same time. In a recent epilepsy machine-learning study, Lu and Triesch (2019) exploited an automated epileptic signal classification method based on deep neural networks with residual connections. To observe the efficacy of their proposed method, two datasets, including Bonn University and the Bern-Barcelona data sets, were used for two kind of separate classification problems in epilepsy, such as the epileptic seizure pattern identification from continuous EEG signals (Bonn University sets) and localization of epileptic focus (Bern-Barcelona data sets). Fraiwan and Alkhodari (2020) used an LSTM model to classify the focal and non-focal epileptic EEG data directly from input. Jukic et al. (2020)compared the effects of the ensemble learning method with different machine learning models for the localization of epileptic focus. Table 7 gives a summary of the previously-described neural network methods.

**Table 7:** Summary of neural network methods

Authors	Feature	Classifier	Dataset	Accuracy
Sui et al. (2019)	STFT	CNN	Barcelona	91.8 %
Subathra et al. (2020)	FWHT	ANN	Barcelona	92.8 %
Siddharth et al. (2019)	SM-SSA	SAE-RBFN	Barcelona	99.11 %
Zhao et al. (2018)	Entropy	CNN	Barcelona	83.0 %
San-Segundo et al. (2019)	FT, WT & EMD	CNN	Barcelona	98.9 %
Gagliano et al. (2019)	Bispectral	LSTM	iEEG.org	86.29 %
Zhao et al. (2021)	Entropy & STFT	FCNN	Barcelona	93.44 %
Daoud and Bayoumi (2019)	DCAE & MLP		Barcelona	93.21 %
Li et al. (2019)	1D-CNN		Barcelona	85.14 %
Lu and Triesch (2019)	CNN		Barcelona	91.8 %
Fraiwan and Alkhodari (2020)	Bi-directional LSTM		Barcelona	99.60 %

**Table 8:** Confusion matrix for a two-class problem

	Predicted positive	Predicted negative
Actual positive	TP: True Positive	FN: False Negative
Actual negative	FP: False Positive	TN: True Negative

## Evaluation criteria

The evaluation criteria are the key factors that measure the efficacy of the computer-aided solutions. For completeness, a brief review of the concepts of evaluation of performance are presented to know what matrices must be considered during evaluation computer-aided solutions to the problem of epileptic focus localization.

### Segment-wise criteria

A long-term EEG recording is usually segmented into short-time epochs to classify focal and non-focal channels. Therefore, for each segment (epoch), typical metrics can be used to evaluate performance. This section summarizes the evaluation metrics used in related works to identify epileptic seizure focus. The most basic performance evaluation metrics used in different epileptic focus detection studies are accuracy, sensitivity, specificity, precision, false discovery rate (FDR), and $F_1$-scores. However, classification accuracy is valid only if the classes are balanced (Fatourechi et al. 2008; Dornhege et al. 2004), which has the same performance for each class. For imbalanced classes, the sensitivity (SEN), specificity (SPE), precision, and fall-out or false-negative rate (FPR) are more informative performance measures (Fatourechi et al. 2008; Dornhege et al. 2004), and can be computed from the confusion matrix. If the classification depends on a continuous parameter, such as threshold, the receiver operating characteristic (ROC) curve and the area under the curve (AUC) are often used. The representation of classification performance can be computed from the confusion matrix, as illustrated in Table 8. Based on that table, the evaluation metrics can be defined as:

- Accuracy (ACC):
  5 $$\begin{aligned} Accuracy=\frac{TP+TN}{TP+FP+TN+FN}\times 100\%, \end{aligned}$$
- Sensitivity (SEN) or recall:
  6 $$\begin{aligned} SEN=\frac{TP}{TP+FN}\times 100, \end{aligned}$$
- Specificity (SPE):
  7 $$\begin{aligned} SPE=\frac{TN}{TN+FP}\times 100, \end{aligned}$$
- Precision or postitive predictive value (PPV):
  8 $$\begin{aligned} Precision=\frac{TP}{TP+FP}\times 100, \end{aligned}$$
- Fall-out or false positive rate (FPR):
  9 $$\begin{aligned} FPR_{nfocal}=\frac{FP}{TN+FP}\times 100, \end{aligned}$$
- and F$_{1}$ score, which is the harmonic mean of preision and sensitivity defined as:
  10 $$\begin{aligned} F_{1}-score=\frac{2}{\frac{1}{Recall}+\frac{1}{precision}}, \end{aligned}$$

where *TP* is the number of correctly-detected focal segments, and *FN* indicates the number of incorrectly-detected focal segments. *TN* is the number of correctly-detected non-focal segments, and *FP* represents the number of incorrectly-detected non-focal segments.

### Electrode-wise evaluation criteria

**Fig. 8:**
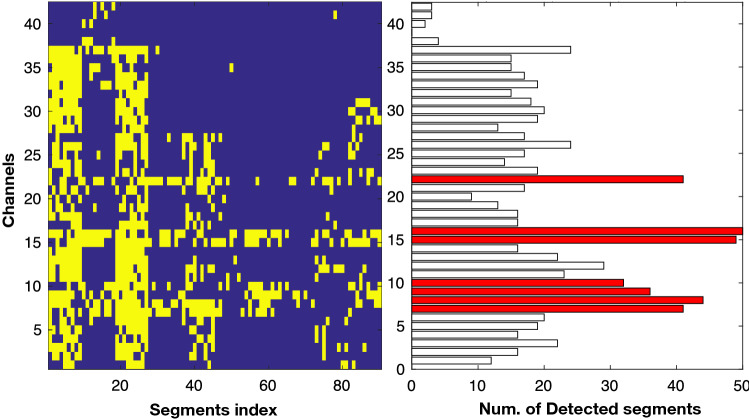
The colormap representation on the left-hand side illustrates the channels (*y*-axis) and segment index (*x*-axis). Each yellow spot in the color map indicates detected focal segments. The right side of the color map (bar) represents the number of detected focal segments (*x*-axis) in each electrode (*y*-axis). The red bars indicate the SOZ channels labeled by epileptologists

Recently, Akter et al. (2020a, 2020b) introduced a score-based evaluation approach in each electrode by counting correctly-detected focal segments, which can be considered as a more effective evaluation criteria for detecting epileptic focus. In conventional clinical systems, epileptologists need to observe long-term multi-channel iEEG signals by dividing them into multiple segments (in their case, 20 s for each segment) to find the epileptic focus. The performance estimation from individual segments does not provide appropriate statistic. Thus, Akter et al. (2020a, 2020b) scored each channel with a number by the correctly-detected focal segments. The SEN and false positive rate (FPR) were estimated for each threshold value from the channel scores. After achieving SEN and PFR with each threshold value, the AUC under receiver operating curve (ROC) was estimated using the trapezoid rule (Fawcett 2006). An example of performance measurement with channels is shown in Fig. 8. It shows an epileptic focus-diagnostic illustration, which can help the epileptologists in two ways: (i) by facilitating observation of the localization of the focal and non-focal segments throughout the multi-channel iEEG signals (as seen on the left side of the Fig. 8). Each yellow spot in the color map represents the detected focal segments; (ii) the number of detected focal segments corresponding to the seizure and non-seizure onset channels (right side of Fig. 8). Figure 8 indicates the number of detected focal segments (x-axis) in each electrode (y-axis) in which the red bars represent the SOZ and the black bars indicates a non-SOZ area. The AUC result for identifying SOZ and non-SOZ channels was estimated for each threshod value from the channel scores shown in right side of Fig. 8. The reader is referred to the work by Akter et al. (2020a, 2020b) on evaluation of computer-aided design for identification of SOZ channels.

## Discussion and open problems

This survey aimed to provide a comprehensive overview of the current epilepsy research focused on developing a computer-aided system for the localization of epileptic focus detection. We have surveyed the different biomarkers- and feature-extraction-based approaches developed between 2013 and 2020 to facilitate creation of an AI-based diagnosis-aid. The numerous explored approaches can be divided into four main categories: (i) HFOs, (ii) PAC, (iii) IED, and (iv) feature-extraction methods.

Some epilepsy studies with a variant of public datasets, including Bonn, Flint Hill, Freiburg, CHB-MIT, Epilepsia, and TUSZ, introduced computer-aided solutions to identify epilepsy patients or seizure patterns in EEG signals. For instance, the Bonn dataset consists of five subjects with single-channel surface EEG at a sampling rate of 173.61 Hz, denoted as Set A (healthy awake and eyes open), Set B (healthy awake and eyes closed), Set C (Epileptic seizure-free interictal), Set D (Epileptic seizure-free interictal), and Set E (epileptic seizure activity). A two-class problem was formed by assigning class labels to epileptic activity (set A) vs others (Set B, C, D, and E). Therefore, different combinations, such as E vs. A, E vs. B, E vs. C, E vs. D, and so on, were performed to detect epileptic vs. non-epileptic activities. However, this dataset’s main goal was to detect epilepsy patients from other subjects. Similarly, the other datasets listed in Table 2 only focused on detecting either seizure or non-seizure patterns in an EEG segment.

For drug-resistant epilepsy, the primary clinical problem is identifying the epileptic focus that must be removed entirely in order to halt seizures. To identify the epileptic focus for surgery, recent neurology hospitals have used grid electrodes (multi-channel) with a high sampling rate to observe ripple (80–250 Hz) and fast ripple (250–600 Hz) bands. The underlying idea behind using a high sampling rate is that ripple and fast ripple bands bear the potential guide for surgical treatment of drug-resistant focal epilepsy. Of note, the epilepsy biomarkers (HFOs, PAC, and IEDs) may occur during ictal, preictal, and interictal states, and the rate of biomarker events tends to be higher in the SOZ channels. As discussed in the biomarker section, many research works based on AI aim to develop automated methods for finding these biomarkers in multi-channel EEG signals. However, the biomarkers in EEGs are differentiated into ripples and fast ripples in EEG signals, and the identification of the biomarkers requires separate ripple and fast ripple iEEGs to identify SOZ channels. When designing a computer-aided solution using these biomarkers, most of the algorithms encounter a common problem: precisely calculating baseline activity. This is because the background activity is not perfectly flat in multi-channel EEG. It is notable that there are several possible subgroups of neurological biomarkers in EEG signals, including HFOs (ripples, fast ripples, and ripples+fast ripples) and IEDs (spikes, wave complex, polyspike, slow-wave, and artifacts). These biomarkers’ variants collectively hint at an intricate AI solution for the identification of possible SOZ channels. However, due to the lack of a standard public dataset related to the biomarkers and their distinctive performance evaluation, it is infeasible to compare the literature’s biomarker-related AI systems.

According to the various studies in this paper, many techniques have introduced the feature-extraction methods described in Table 5 to identify epileptic focus from EEG signals collected in public and hospital datasets. The studies listed in Table 5 collectively provide evidence that the information-theoretic and statistical features are another engineering marker that may help researchers to identify the epileptic focus. Among the many significant advantages of designing a computer-aided system is that it does not need to find a variant of biomarkers in EEG signals, as the statistical features measure the data dispersion from EEG signals to characterize normal and epileptic activities. It is important to note that EEG signals recorded from epilepsy patients may consist of epileptic biomarkers during ictal and interictal phases. The use of statistical methods may provide exciting tools that allow scholars to isolate the normal and epileptic properties from EEG signals and eventually design computer-aided solutions for the identification of SOZ channels.

Based on our survey, we also wish to identify some research problems that must be resolved before more efficient computer-aided solutions can be designed following:

- In our survey, recent studies that utilize engineering solutions to identify SOZ channels have shown promising results. Different ages and pathological types with an increased number of patients should be considered for future studies.
- Most of the studies in this survey focused on developing patient-dependent methods to improve computer-aided systems, not on a patient-independent system (PID). For real-world applications, indeed, the patient-independent design (PID) is preferable because epileptologists require some EEG data to label focal and non-focal electrodes used in the system to hypothesis the possible SOZ channels. However, the design of a patient-independent system for identifying SOZ channels is challenging due to the very different electrodes and subject-specific nature of EEG signals. The most promising directions, those that allow for adaptation to different distributions, could be transfer learning and domain adaptation (Pan and Yang 2009; Lotte and Guan 2010; Azab et al. 2018).
- For designing a supervised computer-aided system (either patient-dependent or patient-independent), the major limitation is the necessity to use SOZ as a prior-basis ground truth for the classifier training stage. Therefore, designing an unsupervised computer-aided system for identification of the SOZ can provide a great facility without prior-basis information of ground truth to take a medical decision.
- Data recording for clinical protocol depends on the patient’s conditions. In particular, it is challenging to collect enough data to apply to machine learning. Data augmentation is another hot topic in AI design, as it could be used to improve system usability and reduce the training set. Some attempts at data augmentation of focus detection have been reported (Akter et al. 2020a, b).
- The selection of influential parameters is another critical factor to the design of a computer-aided system. Parameters with more intelligent signal processing and feature-extraction methods are required to further improve focus identification performance.
- Statistical and information-theoretic features in high-frequency components are promising in this application; however, the interpretation of these features in terms of clinical neurophysiology are still in question.

To conclude, this review suggests that the development of robust, efficient, and convenient computer-aided systems based on feature-extraction methods for identifying SOZ channels provides a new direction in epilepsy research. The development of a trustworthy system remains an open challenge. The next generation of machine-learning approaches to computer-aided diagnosis systems will have to take the user into account. Furthermore, we would like to emphasize that the collaboration between engineering and medical sectors is the most crucial key to the success of a practical computer-aided diagnosis aid.
